# Single-nucleus profiling unveils a geroprotective role of the FOXO3 in primate skeletal muscle aging

**DOI:** 10.1093/procel/pwac061

**Published:** 2022-11-22

**Authors:** Ying Jing, Yuesheng Zuo, Yang Yu, Liang Sun, Zhengrong Yu, Shuai Ma, Qian Zhao, Guoqiang Sun, Huifang Hu, Jingyi Li, Daoyuan Huang, Lixiao Liu, Jiaming Li, Zijuan Xin, Haoyan Huang, Juan Carlos Izpisua Belmonte, Weiqi Zhang, Si Wang, Jing Qu, Guang-Hui Liu

**Affiliations:** State Key Laboratory of Stem Cell and Reproductive Biology, Institute of Zoology, Chinese Academy of Sciences, Beijing 100101, China; University of Chinese Academy of Sciences, Beijing 100049, China; University of Chinese Academy of Sciences, Beijing 100049, China; CAS Key Laboratory of Genomic and Precision Medicine, Beijing Institute of Genomics, Chinese Academy of Sciences, Beijing 100101, China; China National Center for Bioinformation, Beijing 100101, China; Department of Obstetrics and Gynecology, Center for Reproductive Medicine, Peking University Third Hospital, Beijing 100191, China; Clinical Stem Cell Research Center, Peking University Third Hospital, Beijing 100191, China; The Key Laboratory of Geriatrics, Beijing Institute of Geriatrics, Institute of Geriatric Medicine, Chinese Academy of Medical Sciences, Beijing Hospital/National Center of Gerontology of National Health Commission, Beijing 100730, China; Department of Orthopaedics, Peking University First Hospital, Beijing 100034, China; State Key Laboratory of Membrane Biology, Institute of Zoology, Chinese Academy of Sciences, Beijing 100101, China; Institute for Stem Cell and Regeneration, Chinese Academy of Sciences, Beijing 100101, China; Beijing Institute for Stem Cell and Regenerative Medicine, Beijing 100101, China; Advanced Innovation Center for Human Brain Protection, National Clinical Research Center for Geriatric Disorders, Xuanwu Hospital Capital Medical University, Beijing 100053, China; Aging Translational Medicine Center, International Center for Aging and Cancer, Beijing Municipal Geriatric Medical Research Center, Xuanwu Hospital, Capital Medical University, Beijing 100053, China; State Key Laboratory of Stem Cell and Reproductive Biology, Institute of Zoology, Chinese Academy of Sciences, Beijing 100101, China; University of Chinese Academy of Sciences, Beijing 100049, China; State Key Laboratory of Membrane Biology, Institute of Zoology, Chinese Academy of Sciences, Beijing 100101, China; Institute for Stem Cell and Regeneration, Chinese Academy of Sciences, Beijing 100101, China; Beijing Institute for Stem Cell and Regenerative Medicine, Beijing 100101, China; State Key Laboratory of Membrane Biology, Institute of Zoology, Chinese Academy of Sciences, Beijing 100101, China; Institute for Stem Cell and Regeneration, Chinese Academy of Sciences, Beijing 100101, China; Beijing Institute for Stem Cell and Regenerative Medicine, Beijing 100101, China; Advanced Innovation Center for Human Brain Protection, National Clinical Research Center for Geriatric Disorders, Xuanwu Hospital Capital Medical University, Beijing 100053, China; Aging Translational Medicine Center, International Center for Aging and Cancer, Beijing Municipal Geriatric Medical Research Center, Xuanwu Hospital, Capital Medical University, Beijing 100053, China; University of Chinese Academy of Sciences, Beijing 100049, China; CAS Key Laboratory of Genomic and Precision Medicine, Beijing Institute of Genomics, Chinese Academy of Sciences, Beijing 100101, China; China National Center for Bioinformation, Beijing 100101, China; University of Chinese Academy of Sciences, Beijing 100049, China; CAS Key Laboratory of Genomic and Precision Medicine, Beijing Institute of Genomics, Chinese Academy of Sciences, Beijing 100101, China; China National Center for Bioinformation, Beijing 100101, China; Sino-Danish College, University of Chinese Academy of Sciences, Beijing 101408, China; State Key Laboratory of Membrane Biology, Institute of Zoology, Chinese Academy of Sciences, Beijing 100101, China; Institute for Stem Cell and Regeneration, Chinese Academy of Sciences, Beijing 100101, China; Beijing Institute for Stem Cell and Regenerative Medicine, Beijing 100101, China; Advanced Innovation Center for Human Brain Protection, National Clinical Research Center for Geriatric Disorders, Xuanwu Hospital Capital Medical University, Beijing 100053, China; Aging Translational Medicine Center, International Center for Aging and Cancer, Beijing Municipal Geriatric Medical Research Center, Xuanwu Hospital, Capital Medical University, Beijing 100053, China; Altos Labs, Inc., San Diego, CA 94022, USA; University of Chinese Academy of Sciences, Beijing 100049, China; Institute for Stem Cell and Regeneration, Chinese Academy of Sciences, Beijing 100101, China; CAS Key Laboratory of Genomic and Precision Medicine, Beijing Institute of Genomics, Chinese Academy of Sciences, Beijing 100101, China; China National Center for Bioinformation, Beijing 100101, China; Sino-Danish College, University of Chinese Academy of Sciences, Beijing 101408, China; Sino-Danish Center for Education and Research, Beijing 101408, China; Advanced Innovation Center for Human Brain Protection, National Clinical Research Center for Geriatric Disorders, Xuanwu Hospital Capital Medical University, Beijing 100053, China; Aging Translational Medicine Center, International Center for Aging and Cancer, Beijing Municipal Geriatric Medical Research Center, Xuanwu Hospital, Capital Medical University, Beijing 100053, China; The Fifth People’s Hospital of Chongqing, Chongqing 400062, China; State Key Laboratory of Stem Cell and Reproductive Biology, Institute of Zoology, Chinese Academy of Sciences, Beijing 100101, China; University of Chinese Academy of Sciences, Beijing 100049, China; Institute for Stem Cell and Regeneration, Chinese Academy of Sciences, Beijing 100101, China; Beijing Institute for Stem Cell and Regenerative Medicine, Beijing 100101, China; State Key Laboratory of Membrane Biology, Institute of Zoology, Chinese Academy of Sciences, Beijing 100101, China; Advanced Innovation Center for Human Brain Protection, National Clinical Research Center for Geriatric Disorders, Xuanwu Hospital Capital Medical University, Beijing 100053, China; University of Chinese Academy of Sciences, Beijing 100049, China; Institute for Stem Cell and Regeneration, Chinese Academy of Sciences, Beijing 100101, China; Beijing Institute for Stem Cell and Regenerative Medicine, Beijing 100101, China

**Keywords:** single-nucleus RNA sequencing, primate, aging, skeletal muscle, FOXO3

## Abstract

Age-dependent loss of skeletal muscle mass and function is a feature of sarcopenia, and increases the risk of many aging-related metabolic diseases. Here, we report phenotypic and single-nucleus transcriptomic analyses of non-human primate skeletal muscle aging. A higher transcriptional fluctuation was observed in myonuclei relative to other interstitial cell types, indicating a higher susceptibility of skeletal muscle fiber to aging. We found a downregulation of FOXO3 in aged primate skeletal muscle, and identified FOXO3 as a hub transcription factor maintaining skeletal muscle homeostasis. Through the establishment of a complementary experimental pipeline based on a human pluripotent stem cell-derived myotube model, we revealed that silence of FOXO3 accelerates human myotube senescence, whereas genetic activation of endogenous FOXO3 alleviates human myotube aging. Altogether, based on a combination of monkey skeletal muscle and human myotube aging research models, we unraveled the pivotal role of the FOXO3 in safeguarding primate skeletal muscle from aging, providing a comprehensive resource for the development of clinical diagnosis and targeted therapeutic interventions against human skeletal muscle aging and the onset of sarcopenia along with aging-related disorders.

## Introduction

Skeletal muscle is among the largest organs of the body, comprising roughly 40% of total body mass (Frontera and Ochala, 2015). With age, sarcopenia, manifested as loss of skeletal muscle mass and strength, leads to a decline in physical performance and impairs quality of life (Wilkinson et al., 2018; Larsson et al., 2019; Cai et al., 2022). In addition, as an essential endocrine and metabolic organ (Iizuka et al., 2014; Baskin et al., 2015), mounting evidence shows that skeletal muscle aging also contributes to perturbations in metabolic homeostasis probably by changing muscle-derived growth factors (myokines) or metabolites (Tieland et al., 2018; Gomarasca et al., 2020; Wang and Ng, 2022), ultimately increasing the risk of age-associated metabolic disorders such as diabetes and obesity (Kalinkovich and Livshits, 2017; Lipina and Hundal, 2017). Although resistance-type exercise training and diet can help maintain muscle mass (Stokes et al., 2018), effective treatments to delay or prevent skeletal muscle aging and sarcopenia progression in the elderly are still lacking. Therefore, an in-depth understanding of the mechanisms underlying skeletal muscle aging is of scientific and clinical importance.

Skeletal muscle is composed of multinucleated myofibers, embedded mononuclear muscle stem cells (MuSCs), and multiple interstitial cells (Wagers and Conboy, 2005; Frontera and Ochala, 2015). In the shared cytoplasm of muscle fibers, the distribution of diverse myonuclei with heterogeneous gene expression is known to meet functional requirements in distinct regions (Cadot et al., 2015; Orchard et al., 2021). In addition, homeostatic maintenance in skeletal muscle is also regulated by the niche compartment, which includes an array of interstitial cells, such as endothelial cells, smooth muscle cells, fibro-adipogenic progenitors, tendon fibroblasts, and different immune cells (macrophages, etc.) (Giordani et al., 2019; Dos Santos et al., 2020; Kim et al., 2020). The structure and physiology of skeletal muscle become progressively compromised during aging, which is reflected by declined muscle mass and strength at the tissue level (Wilkinson et al., 2018; Simon et al., 2019). At the cellular level, decreases in the number of MuSCs and extent of capillarization, concomitant with defects in neuromuscular junctions (NMJ), as well as a massive accumulation of interstitial adipocytes and infiltration of immune cells were observed with advanced age (Demontis et al., 2013; Livshits and Kalinkovich, 2019; Fuchs and Blau, 2020; Maeda et al., 2021), indicative of both impaired muscle fiber function and dysregulated muscle microenvironment. However, how myonuclei-specific transcriptomic programs shape the architectural and functional changes of multinucleated syncytial muscle cells, and how the interstitial microenvironment orchestrates myofiber deterioration during skeletal muscle aging remain largely unknown.

To develop approaches for treating human skeletal muscle aging and related diseases, it is imperative to gain a comprehensive understanding of human skeletal muscle aging. Despite its importance, ethical restrictions limit the availability of an adequate amount of age-matched skeletal muscle biopsies from healthy donors needed for systematic and in-depth surveys of skeletal muscle aging. As expected from the evolutionary conservation (Anderson and Colman, 2011), non-human primate (NHP) skeletal muscle is highly similar to that of humans in many aspects, including muscle fiber types, neuromuscular junctions, metabolic pathways, and inflammation regulation (Jones et al., 2017; Mercken et al., 2017). Therefore, a study in NHP skeletal muscle aging promises to put forward a better understanding of human skeletal muscle aging, which in turn can help identify biological processes or factors that can be targeted therapeutically.

Here, we generated the single-nucleus transcriptomic atlas of NHP skeletal muscle aging, and identified a panel of nuclear types with distinct and unique gene expression signatures. We also identified and experimentally validated that the downregulation of FOXO3 causally triggered human skeletal muscle aging. Our study provides a valuable resource for understanding the mechanistic underpinning of primate skeletal muscle aging, and facilitates the development of novel therapeutical intervention strategies to combat age-associated muscle degenerative disorders.

## Results

### Characterization of the histological alterations of NHP skeletal muscle aging

**Figure 1:**
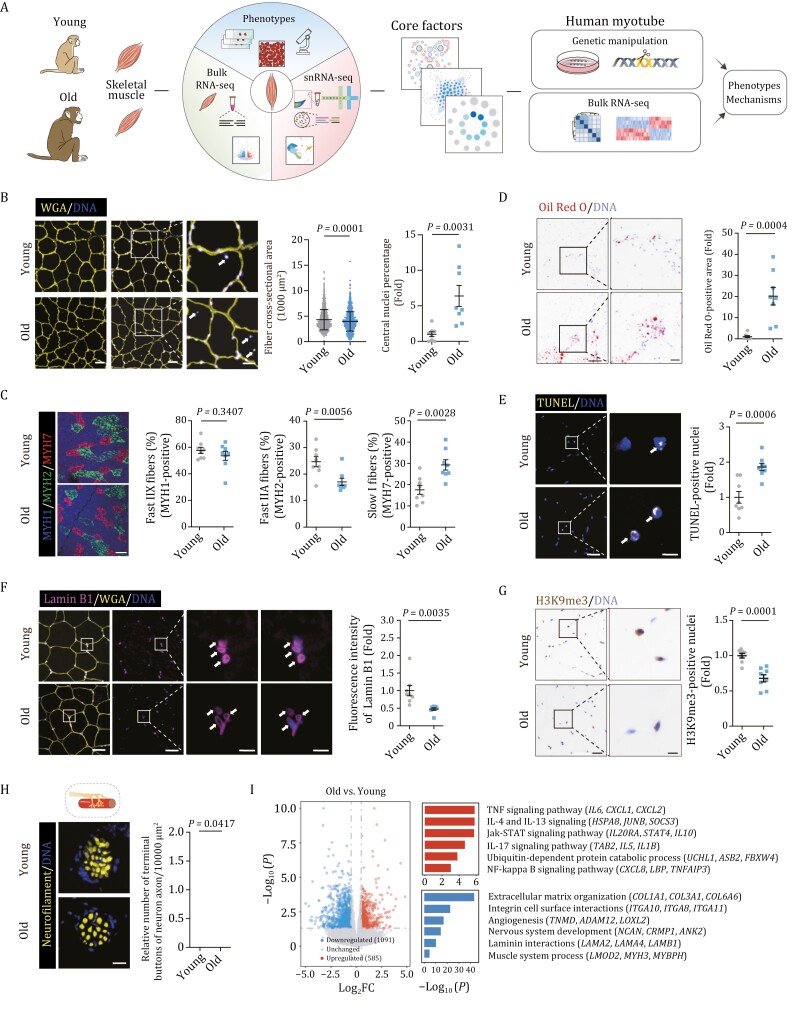
**Characterization of aging-related phenotypes in cynomolgus monkey skeletal muscle.** (A) Workflow showing the procedure of phenotypic analysis, snRNA-seq, bulk RNA-seq of young and old monkey skeletal muscles and functional verification of core factors during aging. (B) Cross-sectional area of muscle fibers and relative central nuclei percentage from young and old monkeys. Representative images are shown on the left. Scale bars, 50 and 25 μm (zoomed-in image). Middle, the quantitative data for the cross-sectional area of myofibers are presented as mean ± SD. *n* = 8 monkeys for each group. Two hundred fibers were calculated for each individual. Right, the percentages of central nuclei were quantified as fold changes in old skeletal muscles vs. in young counterparts and are presented as mean ± SEMs. *n* = 8 monkeys for each group. Arrows indicate central nuclei in skeletal muscle. (C) Cross sections of skeletal muscle stained with antibodies specific for Fast IIX (marked by MYH1), Fast IIA (marked by MYH2), and Slow I fibers (marked by MYH7). Representative images are shown on the left. Scale bar, 100 μm. The percentages of each fiber type were quantified separately as fold changes in old skeletal muscles vs. in young counterparts and are presented as mean ± SEMs on the right. *n* = 8 monkeys for each group. (D) Oil Red O staining of muscle cross sections from young and old monkeys. Scale bars, 200 and 50 μm (zoomed-in image). Oil Red O-positive areas were quantified as fold changes in old skeletal muscles vs. in young counterparts and are presented as mean ± SEMs on the right. *n* = 8 monkeys for each group. (E) TUNEL staining in the skeletal muscles from young and old monkeys. Representative images are shown on the left. Scale bars, 50 and 10 μm (zoomed-in image). The numbers of TUNEL-positive nuclei were quantified as fold changes in old skeletal muscles vs. in young counterparts and are presented as mean ± SEMs on the right. *n* = 8 monkeys for each group. Arrows indicate TUNEL-positive nuclei in skeletal muscle. (F) Lamin B1 immunofluorescence staining in the skeletal muscles from young and old monkeys. Representative images are shown on the left. Scale bars, 50 and 10 μm (zoomed-in image). The fluorescence intensity of Lamin B1 was quantified as fold changes in old skeletal muscles vs. in young counterparts and are presented as mean ± SEMs on the right. *n* = 8 monkeys for each group. Arrows indicate Lamin B1-positive nuclei in skeletal muscle. (G) H3K9me3 immunohistochemical staining in skeletal muscles from young and old monkeys. Representative images are shown on the left. Scale bars, 50 and 25 μm (zoomed-in image). The numbers of H3K9me3-positive nuclei were quantified as fold changes in old skeletal muscles vs. in young counterparts and are presented as mean ± SEMs on the right. *n* = 8 monkeys for each group. (H) Analysis of terminal buttons in the NMJ by immunofluorescence staining of neurofilament in young and old monkeys. Representative images are shown on the left. Scale bar, 25 μm. The numbers of terminal buttons in the NMJ in fixed regions were quantified as fold changes in old skeletal muscles vs. in young counterparts and are presented as mean ± SEMs on the right. *n* = 8 monkeys for each group. (I) Left, volcano plot showing the relative expression levels of DEGs between old and young (O/Y) monkey skeletal muscles. Right, representative Gene Ontology (GO) terms and corresponding DEGs.

To dissect the phenotypic and molecular characteristics of NHP skeletal muscle aging, we obtained skeletal muscle from eight young (4–6 years old) and eight old (18–21 years old) cynomolgus monkeys, which are approximately ~ 16 and ~ 60 years old in human age, respectively (Figs. 1A and S1A) (Wang et al., 2020, 2021b; Li et al., 2021; Ma et al., 2021; Zhang et al., 2021, 2022; Zou et al., 2022). First, we found a decrease in the overall fiber cross-sectional area in aged NHP muscle (Fig. 1B), one of the classic indicators of skeletal muscle aging (Demontis et al., 2013). Additionally, we observed an increment in MYH7-positive type I slow-twitch fiber, while a decline in MYH2-labeled type IIA fast-twitch fiber (Fig. 1C), consistent with the aging-accompanying transition of fiber type (fast-twitch fiber to slow-twitch fiber) (Demontis et al., 2013). Notably, we also observed a panel of classic hallmarks for skeletal muscle aging and degeneration in aged NHP muscle (Dirks et al., 2006; Lipina and Hundal, 2017; Zhao and Chen, 2022; Zhang et al., 2015; 2020b), including an increased percentage of central nuclei in myofibers, accumulated intramuscular lipid deposition, elevated apoptosis, as well as accelerated loss of Lamin B1 and heterochromatin erosion (reflected by decreased H3K9me3 levels) (Fig. 1B and 1D–G). Concurrently, a decreased terminal button in the NMJ was also observed in the skeletal muscle of aged monkey, suggesting defects in neuromuscular junctions in skeletal muscle with advanced age (Fig. 1H). Collectively, histological analysis revealed multifaceted aging-associated damages in aged NHP skeletal muscle.

### Single-nucleus transcriptome profiling of young and old NHP skeletal muscles

To unravel the transcriptomic alterations underlying age-associated changes in NHP skeletal muscle, we performed genome-wide RNA sequencing analysis (Fig. 1I and Table S1). We found that upregulated aging-associated differentially expressed genes (DEGs) were involved in the pro-inflammatory response, including TNF-signaling pathway (*IL6*, *CXCL1*, *CXCL2*) and NF-κB signaling pathway (*CXCL8*, *LBP*, *TNFAIP3*) (Fig. 1I). Importantly, pathway related to the ubiquitin-dependent protein catabolic processes (*UCHL1*, *ASB2*, *FBXW4*) was also activated in the aged skeletal muscle (Fig. 1I). Downregulated DEGs included genes broadly involved in the muscle structure (*LMOD2*, *MYH3*, *MYBPH*) and nervous system development (*NCAN*, *CRMP1*, *ANK2*), in line with the aging phenotypes observed in the aged NHP muscle (Fig. 1B and 1H). Altogether, these findings pinpointed molecular features underlying aging-associated defects in aged NHP skeletal muscle.

**Figure 2.**
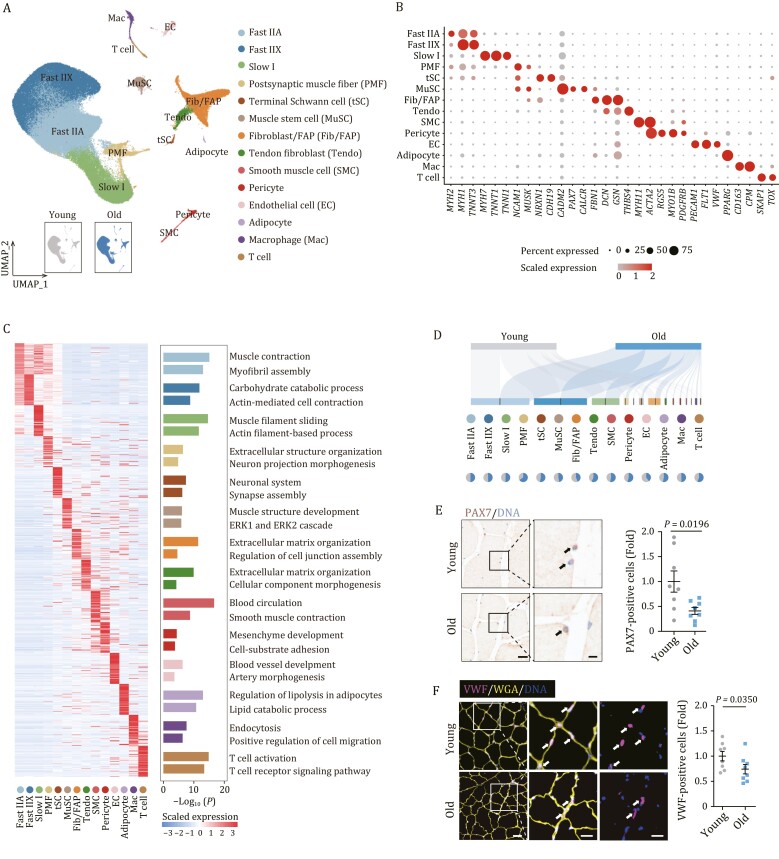
**Single-nucleus transcriptomic atlas of cynomolgus monkey skeletal muscle aging.** (A) Uniform manifold approximation and projection (UMAP) plot showing the 14 cell types of monkey skeletal muscle. Cells are annotated to the right. PMF, postsynaptic muscle fiber; tSC, terminal Schwann cell; MuSC, muscle stem cell; Fib/FAP, Fibroblast/fibro-adipogenic progenitor; Tendo, Tendon fibroblast; SMC, Smooth muscle cell; EC, Endothelial cell; Mac, Macrophage. UMAP plot in the lower left showing the distribution of cells from young and old groups. (B) Dot plot showing the expression signatures of representative marker genes for each cell type. (C) Left, heatmap showing the expression proﬁles of top 50 genes ranked by LogFC of each cell type. Right, enriched GO terms for marker genes of each cell type. (D) Sankey plot showing the distribution of young and aged cells across different cell types. Pie plots showing the relative cell proportion of between old and young groups across different cell types. (E) PAX7 immunohistochemical staining in skeletal muscles from young and old monkeys. Representative images are shown on the left. Scale bars, 20 and 5 μm (zoomed-in image). The numbers of PAX7-positive cells were quantified as fold changes in old skeletal muscles vs. in young counterparts and the quantitative data are presented as mean ± SEMs on the right. *n* = 8 monkeys for each group. Arrows indicate PAX7-positive cells in skeletal muscle. (F) VWF immunofluorescence staining in skeletal muscles from young and old monkeys. Representative images are shown on the left. Scale bars, 50 and 25 μm (zoomed-in image). The numbers of VWF-positive cells were quantified as fold changes in old skeletal muscles vs. in young counterparts and are presented as mean ± SEMs on the right. *n* = 8 monkeys for each group. Arrows indicate VWF-positive cells in skeletal muscle.

To resolve the cell type-specific transcriptional alterations of the aged NHP skeletal muscle, we performed single-nucleus RNA sequencing (snRNA-seq). After stringent filtration, we retained 112,493 qualified single-nucleus transcriptomes from 16 individuals (Fig. S1B–D). Using unbiased clustering and uniform manifold approximation and projection (UMAP) analysis, we identified 14 major populations in skeletal muscle with distinct transcriptomic signatures (Fig. 2A and Table S2). Among these, we identified four types of myonuclei from myofibers based on canonical markers, including Slow I, Fast IIA, Fast IIX, and postsynaptic endplates (postsynaptic muscle fiber, PMF), which is known for functional specialization at NMJ (Figs. 2A and S1E–G). In addition, based on the expression of classical marker genes, we annotated MuSC and a total of nine non-myonuclear cell types, including EC, SMC, pericyte, tendon fibroblast (Tendo), fibroblast/FAP (Fib/FAP), adipocyte, terminal Schwann cell (tSC), and two immune cell types (macrophage (Mac) and T cell) (Fig. 2A and 2B). In functional enrichment analysis of the top 50 marker genes of each cell type, we further revealed the transcriptional characteristics related to their unique physiological functions (Fig. 2C). For example, the marker genes of nuclei from Fast IIA, Fast IIX, and Slow I muscle fiber aligned with muscle functions, such as muscle contraction and muscle filament sliding, whereas those of PMF and tSC aligned with neuron projection morphogenesis and neuronal system (Fig. 2C). Consistent with the compromised muscle regenerative capacity in the elders (Larsson et al., 2019), we found the number of PAX7-positive MuSCs declined with age, indicative of attrition of the stem cell pool (Figs. 2D, 2E, and S2A). Concurrently, in the aged skeletal muscle, we noticed a prominent reduction in cell number of EC relative to young counterparts (Figs. 2D, 2F, and S2B), indicative of a dysregulated interstitial microenvironment in aged muscle.

### Cell type-specific alterations in gene expression programs of NHP skeletal muscle aging

**Figure 3.**
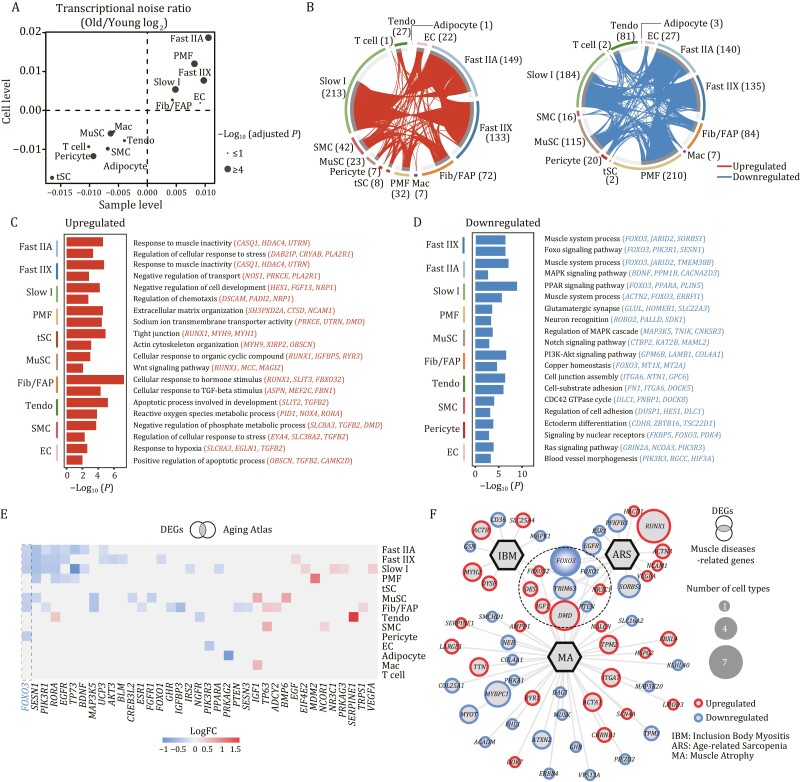
**Cell type-specific transcriptional alterations during cynomolgus monkey skeletal muscle aging.** (A) Scatter plot showing the log_2_ ratio of transcriptional noise between old and young samples as calculated using sample averages (*n* = 16) and single cells on the X and Y axes, respectively. (B) Circos plots showing the aging-related differentially expressed genes (O/Y) DEGs, adjusted *P* values < 0.05 and |LogFC| > 0.25 for each cell type between old and young groups. Each connecting curve represents a gene up- or downregulated in two cell types. The number of DEGs for each cell type is also annotated. (C and D) Bar charts showing the enriched GO terms for upregulated (C) or downregulated (D) DEGs between old and young groups across different cell types in monkey skeletal muscle. (E) Heatmap showing the DEGs included in Aging Atlas database. (F) Network plot showing the DEGs associated with aging-related muscle diseases. The node size of genes indicates the number of cell types in which this gene was differentially expressed with age. The genes in the circle with a dashed line are associated with at least two kinds of diseases.

Given that increased transcriptional heterogeneity and perturbance are regarded as one of the features of mammalian aging (Wang et al., 2020; Li et al., 2021; Ma et al., 2021; Zhang et al., 2021; Leng and Pawelec, 2022; Zou et al., 2022), we sought to assess transcriptional noise during NHP skeletal muscle aging. A calculation of age-associated coefficient of variation demonstrated that aged Fast IIA, Fast IIX, Slow I, and PMF, the major components of skeletal muscle, exhibited a higher cell-to-cell transcriptional noise relative to other populations (Fig. 3A), suggesting a higher susceptibility of skeletal muscle fiber to aging.

We next analyzed aging-associated DEGs (averaged Log|FoldChange| > 0.25 and adjusted *P* values < 0.05) between old (O) and young (Y) groups across different populations (Figs. 3B, S2C, and Table S1). We found that the majority of upregulated DEGs converged in Slow I, Fast IIA, and Fast IIX (213, 149, and 133 genes, respectively) (Fig. 3B), while the majority of downregulated DEGs were identified in PMF, Slow I, Fast IIA, and Fast IIX (210, 184, 140, and 135 genes, respectively) (Fig. 3B), again implying the sensitivity of myofibers to aging. We noticed a prominent upregulation of genes associated with response to muscle inactivity in fast-twitch myonuclei (Fig. 3C), in accordance with the loss of muscle mass and strength that is observed during aging (sarcopenia) and muscle disuse. This upregulation was presented in both Fast IIA and Fast IIX, but not in Slow I (Fig. 3C), consistent with the early onset of muscle degeneration in fast-twitch fiber (Demontis et al., 2013; Murgia et al., 2017). Interestingly, we detected upregulation of genes involved in Wnt signaling pathway in MuSC (Fig. 3C), which has been reported to correlate with the bias in cell fate determination, that is, the transition from a myogenic to a fibrogenic lineage (Domingues-Faria et al., 2016). These data were consistent with an age-dependent loss of the MuSC pool, concordant with the previously observed phenotype (Fig. 2E). Downregulated DEGs in Fast IIA, Fast IIX, and Slow I were consistently related to the maintenance of muscle structure and function (Fig. 3D), suggesting a coordinated muscle dysfunction at advanced age among different fibers. In addition, downregulated genes related to the Notch signaling pathway in MuSC further underlined the age-dependent depletion of MuSC (Fig. 3D) (Jiang et al., 2014; Verma et al., 2018). Further, we found downregulation of genes associated with glutamatergic synapse and neuron recognition in PMF (Fig. 3D), which was in concert with age-related defects in neuromuscular junctions as previously observed (Fig. 1H). Overall, these analyses deciphered the cellular and molecular programs underpinning NHP skeletal muscle aging.

Next, we identified a total of 39 DEGs that were shared in at least five cell types, of which 9 genes were consistently upregulated and 30 genes were consistently downregulated (Fig. S2C). Globally, multiple dysregulated genes were predominantly found in myofibers (Fig. S2C), strengthening the notion that muscle fibers were most responsive to aging. Amongst cell type-specific DEGs were genes that regulate corresponding cellular functions (Fig. S2D). Further, we conducted an integrated analysis of aging-associated DEGs and genes from the Aging Atlas database to identify the expression alterations of genes associated with aging (Guang-Hui Liu, 2021). Among these, *FOXO3* exhibited the most prominent changes, whose downregulation was found in more than five cell types (Fig. 3E). *FOXO3*, encoding a member of the forkhead transcription factor (TF) family, is a well-known longevity gene (Morris et al., 2015; Zhang et al. 2020a; Yan et al. 2019). Its downregulation is likely correlated to the onset of skeletal muscle aging and degeneration. We then asked if these aging-associated DEGs are linked to degenerative diseases in skeletal muscle. To this end, we performed a joint comparative analysis of aging-associated DEGs and hotspot genes known to be involved in age-related muscle diseases (sarcopenia, muscle atrophy, and inclusion body myositis) (Navarro, 2001; Askanas and Engel, 2007; Yu et al., 2010; Askanas et al., 2012; González et al., 2015; Wilkinson et al., 2018) (Figs. 3F, S2E and Table S3). Interestingly, we noticed that *FOXO3* and its homologous gene *FOXO1*, as the prominent hub genes, were closely linked to various kinds of age-related muscle disorders (Fig. 3F), suggesting a putative crucial role of FOXO family in the regulation of NHP skeletal muscle aging.

### Transcriptional regulatory network pinpoints FOXO3 as a core transcription factor during NHP skeletal muscle aging

**Figure 4.**
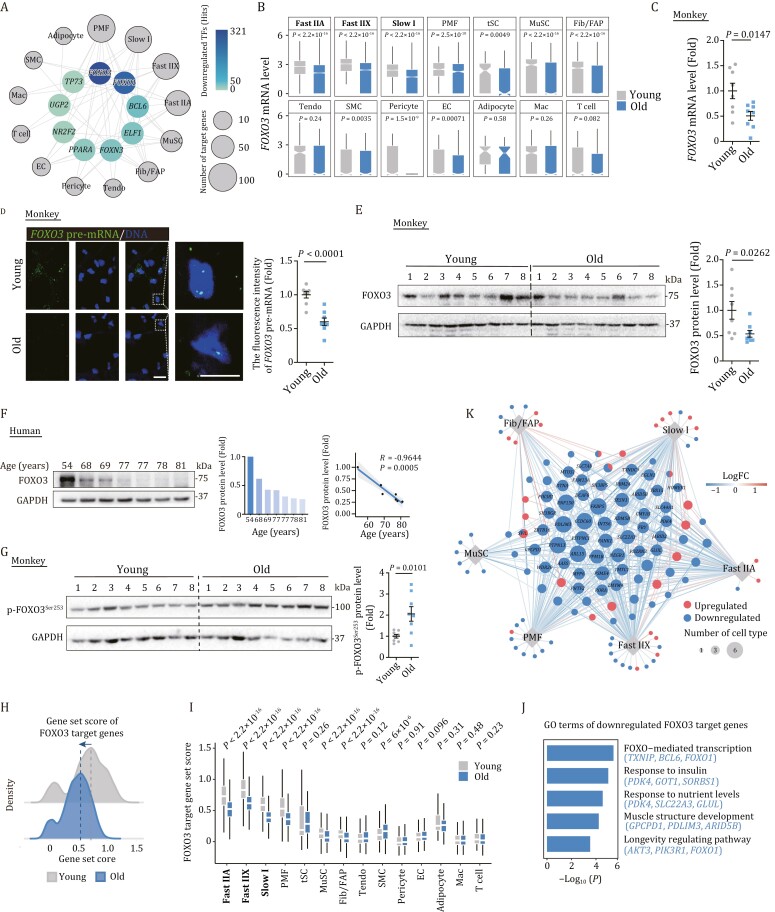
**Transcriptional network pinpoints FOXO3 as a crucial transcription factor in regulating primate skeletal muscle aging.** (A) Network visualization of downregulated core transcriptional regulators in different cell types between old and young groups. Outer nodes display different cell types and the node size positively correlates with the number of target genes differentially expressed in corresponding cell types. (B) Box plots showing *FOXO3* mRNA expression level across different cell types in monkey skeletal muscles from old and young groups. (C) RT-qPCR analysis showing the *FOXO3* mRNA expression changes in young and old monkey skeletal muscles. *FOXO3* mRNA levels were quantified as fold changes (old vs. young), and are presented as mean ± SEMs. *n* = 8 monkeys for each group. (D) RNA-FISH assay showing the *FOXO3* expression changes in young and old monkey skeletal muscle tissues. Representative images are shown on the left. Scale bars, 20 and 10 μm (zoomed-in image). Right, fluorescence intensity of *FOXO3* pre-mRNA level was quantified as fold changes (old vs. young) and are presented as mean ± SEMs. *n* = 8 monkeys for each group. (E) Western blot and band intensity quantification of FOXO3 protein levels in young and old monkey skeletal muscle samples. Data were quantified as fold changes in old skeletal muscles vs. in young counterparts and are presented as mean ± SEMs. *n* = 8 monkeys for each group. (F) Left, western blot and band intensity quantification of FOXO3 protein levels in human skeletal muscle samples. GAPDH was used as the loading control. Middle, bar plot showing the relative expression of FOXO3 protein levels for each individual. Right, the negative correlation of relative FOXO3 protein levels in skeletal muscle across different ages. The shadow indicates the 0.95 confidence interval around smooth. *n* = 7 donors. (G) Western blot and band intensity quantification of phospho-FOXO3 protein levels in young and old monkey skeletal muscle samples. Data were quantified as fold changes in old skeletal muscles vs. in young counterparts and are presented as mean ± SEMs. *n* = 8 monkeys for each group. (H) Ridge map showing the global distribution density of gene set score of FOXO3 target genes identified by SCENIC. The corresponding dashed line represents the peak position of each group. (I) Box plot showing the gene set score of FOXO3 target genes across each cell type of monkey muscles from old and young groups. (J) Bar plot showing representative GO terms of downregulated FOXO3 target genes. (K) Network visualization of FOXO3 target genes in Slow I, Fast IIA, Fast IIX, PMF, MuSC, and Fib/FAP cell types. Node size positively correlates with the number of cell types. Each connecting line represents gene differentially expressed in the corresponding cell type.

To further explore the core TFs governing aging-associated DEGs, we constructed a transcriptional regulatory network through Single-Cell Regulatory Network Inference and Clustering (SCENIC) analysis (Figs. 4A, S2F, and Table S4). Of note, we identified FOXO3 as one of the top downregulated transcriptional regulators in aged skeletal muscle (Fig. 4A). Indeed, *FOXO3* transcripts were downregulated in the majority of cell types in aged skeletal muscle, along with a reduction in protein levels of FOXO3 in aged NHP muscle tissues as an entirety (Figs. 4B–E and S2G). Interestingly, we also found an age-dependent decline of FOXO3 protein in human skeletal muscle (Fig. 4F). More strikingly, phosphorylated FOXO3, an inactive form of this protein, that tends to be excluded from the nucleus and undergoes consequent ubiquitination and degradation (Sandri et al., 2004; Zhao et al., 2007; Yan et al., 2019), was elevated in the aged NHP muscle (Fig. 4G).

We next investigated the transcriptional activity of the FOXO3-modulated regulatory network (Calissi et al., 2021), and found that the scores of predicted FOXO3-target genes were lower in aged skeletal muscle and particularly in each fiber type relative to their young counterparts (Fig. 4H, 4I and Table S3). Furthermore, the downregulated FOXO3 target genes were relevant to longevity pathways (*AKT3*, *PIK3R1*, *FOXO1*) and muscle structure development or homeostasis maintenance (*GPCPD1*, *PDLIM3*, *ARID5B*) (Fig. 4J). For instance, *PDLIM3* encodes cytoskeletal-related protein colocalizing with alpha-actinin-2 at the Z lines of skeletal muscle and is involved in cytoskeletal assembly (Ohsawa et al., 2011), whose downregulation may lead to a pronounced instability in skeletal muscle structure (Fig. 4K). The aforementioned results suggested that the inactivation of FOXO3 and its downstream cascade may underlie NHP skeletal muscle degeneration.

### *FOXO3* acts as a major effector gene to protect skeletal muscle from aging

**Figure 5.**
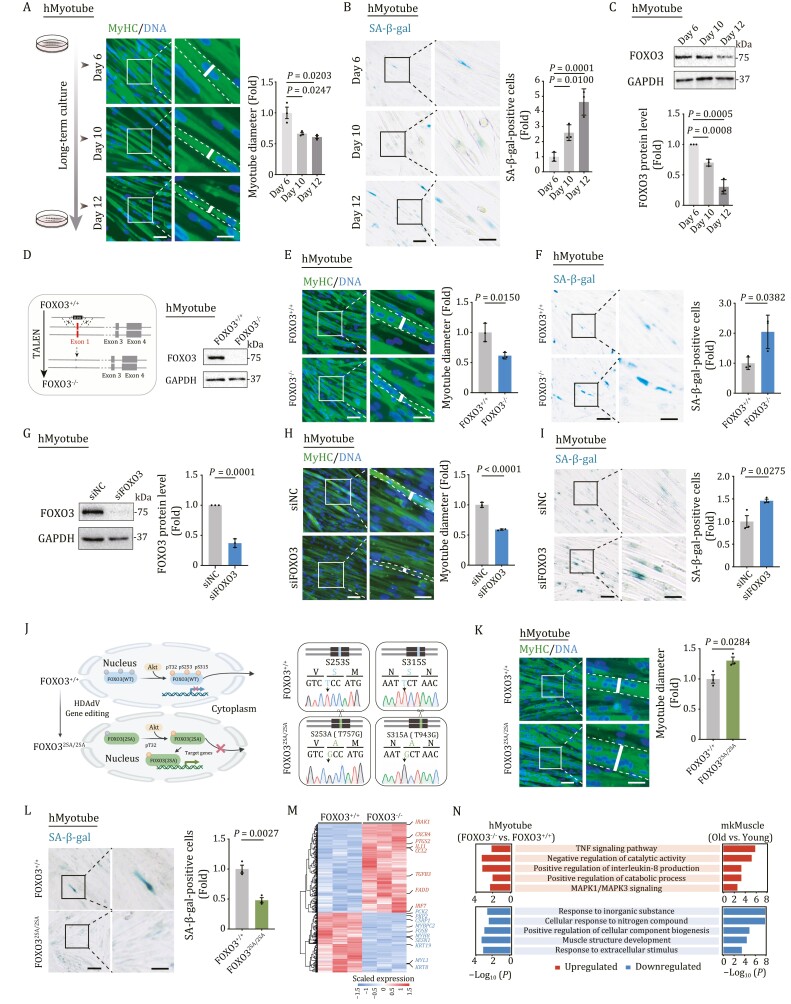
**FOXO3 protects human myotubes from senescence.** (A) Left, representative immunofluorescence images of MyHC-positive hMyotube after long-term culture for 6, 10, and 12 days. Scale bars, 50 and 25 μm (zoomed-in image). Right, the diameters of the hMyotubes were quantified as fold changes in hMyotubes at Day 10 or Day 12 vs. at Day 6, and are shown as mean ± SEMs. *n* = 3 biological replicates. (B) Left, representative images of SA-β-gal-positive cells of WT hMyotubes after a long-term culture for 6, 10, and 12 days. Scale bars, 100 and 50 μm (zoomed-in image). Right, SA-β-gal-positive cells of the myotubes were quantified as fold changes in hMyotubes at Day 10 or Day 12 vs. at Day 6, and are presented as mean ± SEMs. *n* = 3 biological replicates. (C) Western blot analysis showing the protein levels of FOXO3 in WT hMyotubes after a long-term culture for 6, 10, and 12 days. GAPDH was used as the loading control. Band intensity were quantified as fold changes in hMyotubes at Day 10 or Day 12 vs. at Day 6 and are presented as mean ± SEMs. *n* = 3 independent experiments. (D) Left, schematic showing siRNA-mediated knockdown of *FOXO3* in WT hMyotubes. Right, Western blot analysis of FOXO3 protein expression in FOXO3^+/+^ and FOXO3^−/−^ hMyotubes. GAPDH was used as the loading control. (E) MyHC immunofluorescence staining in FOXO3^+/+^ and FOXO3^−/−^ hMyotubes. Representative images are shown on the left. Scale bars, 50 and 25 μm (zoomed-in image). Right, the diameters of the hMyotubes were quantified as fold changes (FOXO3^−/−^ vs. FOXO3^+/+^) and are presented as mean ± SEMs. *n* = 3 biological replicates. (F) Left, representative SA-β-gal staining images of FOXO3^+/+^ and FOXO3^−/−^ hMyotubes. Scale bars, 100 and 50 μm (zoomed-in image). Right, SA-β-gal-positive cells of the myotubes were quantified as fold changes (FOXO3^−/−^ vs. FOXO3^+/+^) and are presented as mean ± SEMs. *n* = 3 biological replicates. (G) Western blot showing the protein levels of FOXO3 in hMyotubes upon the knockdown of *FOXO3*. GAPDH was used as the loading control. Band intensity were quantified as fold changes (si-FOXO3 vs. si-NC) and are presented as mean ± SEMs. *n* = 3 independent experiments. (H) MyHC immunofluorescence staining of the hMyotubes transfected with si-NC or si-FOXO3. Representative images are shown on the left. Scale bars, 50 and 25 μm (zoomed-in image). The diameters of hMyotubes were quantified as fold changes (si-FOXO3 vs. si-NC) and are presented as mean ± SEMs on the right. *n* = 3 biological replicates. (I) SA-β-gal-positive cells of the hMyotubes transfected with si-NC or si-FOXO3. Representative images are shown on the left. Scale bars, 100 and 50 μm (zoomed-in image). Data were quantified as fold changes (si-FOXO3 vs. si-NC) and are presented as mean ± SEMs on the right. *n* = 3 biological replicates. (J) Left, the schematic shows that edited FOXO3 [FOXO3(2SA)] cannot be phosphorylated by AKT and is constitutively activated in the nucleus. Right, DNA sequencing demonstrates base conversion in FOXO3^2SA/2SA^ hMyotubes. The base conversion of T757G and T943G in genomic DNA results in the change of S253A and S315A in the protein sequence, respectively. (K) MyHC immunofluorescence staining in FOXO3^+/+^ and FOXO3^2SA/2SA^ hMyotubes. Representative images are shown on the left. Scale bars, 50 and 25 μm (zoomed-in image). The diameters of hMyotubes were quantified as fold changes (FOXO3^2SA/2SA^ vs. FOXO3^+/+^) and are presented as mean ± SEMs on the right. *n* = 3 biological replicates. (L) Left, representative SA-β-gal staining images of FOXO3^+/+^ and FOXO3^2SA/2SA^ hMyotubes. Scale bars, 100 and 50 μm (zoomed-in image). Right, SA-β-gal-positive myotubes were quantified as fold changes (FOXO3^2SA/2SA^ vs. FOXO3^+/+^) and are presented as mean ± SEMs. *n* = 3 biological replicates. (M)Heatmap showing the expression levels of DEGs between FOXO3^−/−^ and FOXO3^+/+^ hMyotubes based on RNA-seq analysis. (N) GO terms shared by aging-associated DEGs in monkey skeletal muscle (mkMuscle) and DEGs between FOXO3^−/−^ and FOXO3^+/+^ hMyotubes.

To investigate the function and molecular mechanism of FOXO3 in human skeletal muscle aging, we first generated human myotube (hMyotube) via directed differentiation from human embryonic stem cell (hESC) in a culture dish (Maffioletti et al., 2015). Prolonged culturing of human myotubes *in vitro* can result in a range of aging-related phenotypes, including decreased myotube diameter and elevated senescence-associated β-galactosidase (SA-β-gal) activity (Fig. 5A and 5B). Furthermore, the expression level of FOXO3 in hMyotubes was downregulated with prolonged *in vitro* culture (Fig. 5C), consistent with what we observed in the aged primate skeletal muscle (Fig. 4E and 4F). To investigate the effects of FOXO3 deficiency on hMyotube homeostasis, we generated FOXO3 knockout (FOXO3^−/−^) hMyotubes using TALEN-mediated gene editing technology (Fig. 5D) (see Methods section) (Zhang et al., 2020a). FOXO3^−/−^ hMyotubes underwent accelerated aging, mainly characterized by reduced myotube diameter and increased SA-β-gal activity (Figs. 5E, 5F, and S3A). Similarly, siRNA-mediated knockdown of *FOXO3* mimicked the human myotube aging phenotypes we observed in the FOXO3-deficient hMyotubes (Figs. 5G–I and S3B). Most importantly, we found that the aging phenotypes in hMyotubes were alleviated by gene editing-based activation of endogenous FOXO3 via alanine substitution on two of the three classical phosphorylation sites and generation of a constitutively active version of FOXO3 [FOXO3(2SA)] (Yan et al., 2019), as evidenced by an increment in human myotube diameter and concomitant reduction of SA-β-gal activity (Figs. 5J–L and S3C). When we performed genome-wide RNA sequencing, we found that FOXO3 deficiency in hMyotubes led to gene expression changes resembling those we had observed in skeletal muscle from aged monkeys (Figs. 5M, 5N, S3D, S3E, and Table S1). These included upregulated genes involved in the TNF-signaling pathway and interleukin-8 production, indicative of augmented age-associated inflammation. Upregulated DEGs also commonly converged on positive regulation of catabolic processes (Fig. 5N), suggesting that catabolism is favored upon FOXO3 ablation. Dampened transcriptional programs were commonly linked with muscle structure (Fig. 5N), supporting that FOXO3 downregulation underlies dysregulations of primate skeletal muscle homeostasis and degeneration. Taken together, these data suggest that FOXO3 plays a pivotal role in safeguarding primate muscle integrity against aging.

## Discussion

**Figure 6.**
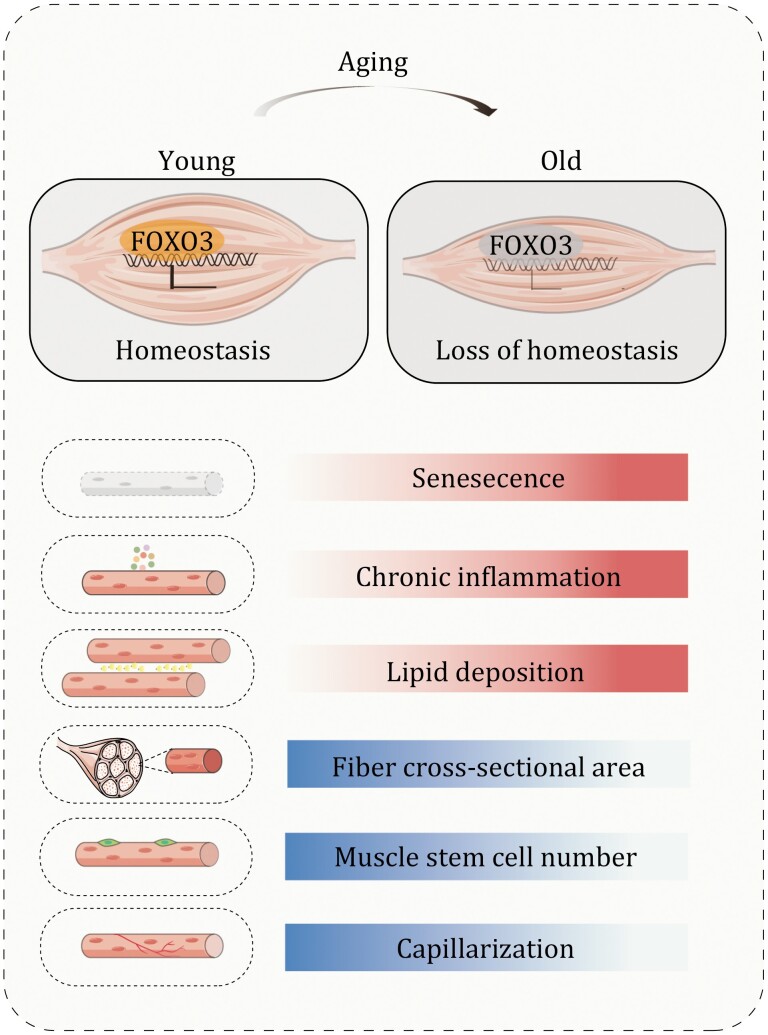
**Working model.** A schematic illustration showing the phenotypic and transcriptomic signatures of primate skeletal muscle aging.

Age-related decline in skeletal muscle mass and strength is a hallmark feature of sarcopenia (McKellar et al., 2021). As such, this decline compromises physical performance and augments the risk of metabolic disease in the elderly. Here, we systemically analyzed aging-related phenotypes and established a single-nucleus transcriptome atlas of primate skeletal muscle aging. These efforts led to the identification of FOXO3 as a protective gatekeeper against primate skeletal muscle aging, and therefore pave the way for the development of novel diagnostics and intervention therapies for human skeletal muscle aging (Fig. 6).

While a few studies captured non-myofiber mononuclear cells by single-cell RNA sequencing (scRNA-seq) (Dell’Orso et al., 2019; Giordani et al., 2019; De Micheli et al., 2020; He et al., 2020; Rubenstein et al., 2020; Limbad et al., 2022), the myofiber syncytium does not easily lend itself to single-cell analyses using conventional dissociation approaches. To overcome this obstacle, we employed snRNA-seq analysis of primate skeletal muscle nuclei in suspension to generate, for the first time, a comprehensive transcriptomic roadmap of physiological private muscle aging. Furthermore, to deepen explorations of molecular events, we complemented our single-nucleus transcriptome profiles with a human pluripotent stem cell-derived myotube model that permits genetic manipulations. Using this combined research platform, we demonstrated a geroprotective role of FOXO3 in primate skeletal muscle aging.

As one of the most conserved and widely recognized human longevity genes, the cytoprotective effect of FOXO3 on various tissues has been widely reported, and FOXO3 is therefore a key target for the prevention and treatment of aging-related diseases (Salih and Brunet, 2008; Yan et al., 2019; Zhang et al., 2020a; Calissi et al., 2021; Lei et al., 2021). Indeed, supporting our observations in aged primate skeletal muscle and prolonged cultured senescent human myotubes, several previous findings demonstrated decreased *FOXO3* mRNA and total or nuclear FOXO3 (active form) protein levels in aged human skeletal muscle (Leger et al., 2008; Dalle and Koppo, 2021). Although mouse-based pioneering studies suggested a deleterious role for FOXO3 in regulating skeletal muscle homeostasis (Sandri et al., 2004; Mammucari et al., 2007; Zhao et al., 2007), the function of FOXO3 during primate skeletal muscle aging remains unexplored. In particular, identifying the causal mechanism underlying primate skeletal muscle aging is challenging, given the lack of a suitable research system. It is for this reason that, in the current study, leveraging extensive mechanistic studies using engineered human myotubes, we for the first time reveal that activation of FOXO3 alleviates its senescence, suggesting an unappreciated protective role of FOXO3 against progressive primate skeletal muscle degeneration.

In conclusion, we here established a comprehensive single-nucleus transcriptomic landscape of primate skeletal muscle aging, which helped identify the inactivation of FOXO3 as a novel biomarker and a potential driver for primate skeletal muscle aging. The single-cell atlas of primate skeletal muscle and the human myotube aging research platform produced by this study promisingly support further explorations of additional diagnostic biomarkers of human skeletal muscle aging and the development of novel therapeutic interventions to treat aging-associated muscle diseases.

## Materials and methods

### Ethical statement

This study was conducted following the guidelines for the Ethical Treatment of nonhuman primates and was approved by the Institutional Animal Care and Use Committee of the Institute of Zoology (Chinese Academy of Sciences). All animal experiments in this study have been approved by the Ethics Review Committee of the Institute of Zoology of the Chinese Academy of Sciences. The collection and use of human skeletal muscle in this study were performed under the approval of the Research Ethics committee of the Peking University First Hospital.

### Experimental animals

Eight young monkeys (4–6 years old) and eight old monkeys (18–21 years old) originated from Southeast Asia were raised at 25°C, 12 h light and dark cycle, and fed a normal diet at a certified Primate Research Center in Beijing (Xieerxin Biology Resource) (Wang et al., 2020; Zhang et al., 2020a, 2021; 2022; Li et al., 2021; Ma et al., 2021). All animals have no clinical or experimental history potentially affecting the physiological aging process.

### Cell culture

Human embryonic stem cells (hESCs, Line H9, from WiCell Research) were maintained on mitomycin C-inactivated Mouse Embryonic Fibroblasts in hESC culture medium (Hu et al., 2020) containing 80% DMEM/F12 (Gibco), 20% Knockout Serum Replacement (Gibco), 2 mmol/L GlutaMAX (Gibco), 0.1 mmol/L nonessential amino acids (NEAA, Gibco), 55 μmol/L β-mercaptoethanol (Invitrogen), and 10 ng/mL bFGF (Joint Protein Central). hESCs were also cultured on Matrigel (BD Biosciences) with mTeSR medium (STEMCELL Technologies) at 37°C with 5% CO_2_. Human myotubes were cultured in high-glucose DMEM medium (Hyclone) containing 2% horse serum (Gibco) and 2 mmol/L GlutaMAX (Gibco) at 37°C with 5% CO_2_ and 3%–5% O_2_ (Maffioletti et al., 2015). HEK293T cells were cultured in high-glucose DMEM (Hyclone) supplemented with 10% fetal bovine serum (FBS, Gibco) at 37°C with 5% CO_2_. There was no mycoplasma contamination observed during cell culture.

### Tissue sampling

Monkey quadriceps muscle tissues were harvested as previously described (Zhang et al., 2018; Liu et al., 2022). Afterwards, the attached fat or fascia tissues were carefully removed. The tissues were then fixed in 4% paraformaldehyde (PFA) at 4°C and embedded in paraffin or directly embedded in Tissue-Tek O.C.T compound (Sakura Finetek) and frozen in liquid nitrogen for histological analysis. The remaining tissues were stored in liquid nitrogen until the nuclei were extracted for sequencing analysis as well as for other RNA and protein analyses. Human quadriceps muscle samples were obtained and washed twice in pre-cold PBS and immediately frozen and stored in liquid nitrogen for further analyses.

### Oil Red O staining

Oil Red O staining was performed as previously described (Ma et al., 2020). In brief, a Leica CM3050S cryostat was used to perform 10-μm-thick cryosections of monkey skeletal muscle embedded in Tissue-Tek O.C.T compound. After air-drying, the sections were fixed in a solution containing 4% PFA at room temperature (RT) for 10 min, and then stained in a freshly prepared Oil Red O staining solution (Sigma-Aldrich) for 30 min, rinsed with tap water and counterstained with hematoxylin. Images were taken with PerkinElmer Vectra Polaris. Image J was used to quantify the percentage of Oil Red O-positive area.

### Immunofluorescence staining

Immunofluorescence staining was performed according to the previous method with minor modifications (Wang et al., 2020). For tissues, muscle tissue was embedded in Tissue-Tek O.C.T compound by the Leica CM3050S cryostat. The 10-μm cryosections were air-dried for 15 min, and then washed three times in PBS. Next, the sections were fixed with 4% PFA for another 20 min, and permeabilized with 0.4% Triton X-100 in PBS for 1 h, and again rinsed in PBS three times. Human myotubes were fixed with 4% PFA in PBS for 20 min and rinsed with PBS twice, and permeabilized with 0.4% Triton X-100 (Sigma-Aldrich) for 1 h at RT. The sections and cells were incubated with blocking buffer (10% donkey serum in PBS) for 1 h at RT and then with primary antibodies overnight at 4°C. After washing several times with PBS, the samples were incubated with fluorescently labeled secondary antibodies for 1 h at RT. The nuclei were stained with Hoechst 33342 (Thermo Fisher Scientific), and the sections were washed three times in PBS and then mounted in VECTERSHIELD anti-fading mounting medium (Vector Laboratories). Images were acquired using a confocal laser-scanning microscope (Leica TCS SP5 II). MyHC (myosin heavy chain)-positive cell containing at least three nuclei and displaying a tube-like structure was defined asa myotube. The diameters of 30–50 myotubes from randomly selected fields in each well were measured using the Image J software. The average diameter of randomly selected myotubes in each well was used for statistical analysis, and there are in total three wells as biological replicates (*n* = 3) (Liu et al., 2018). The antibodies used for immunofluorescence analyses are listed in Table S5.

### Immunohistochemistry staining

Immunohistochemistry staining was performed as previously described with minor modifications (Zou et al., 2021). Briefly, the paraffin-embedded sections were deparaffinized and rehydrated using xylene and graded ethanol. Antigen retrieval was performed by steaming in citrate buffer four times for 2 min each. Sections were rinsed three times in PBS after being slowly cooled down to RT, and then permeabilized with 0.4% Triton X-100 in PBS for 1 h. And then sections were incubated with 3% H_2_O_2_ for 10 min to inactivate endogenous peroxidase and blocked with 10% donkey serum in PBS for 1 h. Sections were then incubated with primary antibodies at 4°C overnight and HRP-conjugated secondary antibodies at RT for 1 h the next day. Sections were performed using the DAB Staining Kit (ZSGB-BIO) according to the manufacturer’s instructions. Finally, sections were dehydrated in a series of graded alcohols (50%, 70%, 80%, 90%, 100%, and 100%) and xylene before being mounted in the neutral resinous mounting medium. Images were taken with PerkinElmer Vectra Polaris. The antibodies used in this study are listed in Table S5.

### Lentivirus packaging

For lentivirus packaging, HEK293T cells were co-transfected with lentiviral vectors, psPAX2 (Addgene) and pMD2G (Addgene) using Lipofectamine 3000 Transfection Reagent (Thermo Fisher Scientific). The supernatants containing viral particles were collected at 48 and 72 h after transfection for ultracentrifugation at 19,400 ×*g* at 4°C for 2.5 h.

### Generation of FOXO3-knockout and FOXO3-enhanced human myotube (hMyotube)

Knockout of FOXO3 in human embryonic stem cells (hESCs) was performed by TALEN-based homologous recombination as previously reported (Zhang et al., 2020a). Genetic activation of *FOXO3* in hESCs was performed by helper-dependent adenoviral vector (HDAdV) for *FOXO3*^*2SA*/*2SA*^ (Ser253Ala and Ser315Ala) knock-in as previously described (Yan et al., 2019). Human myotubes were then derived from the *FOXO3*-deficient and *FOXO3*-activated hESCs using a previously published protocol with some modifications (Maffioletti et al., 2015). In brief, hESCs were cultured in differentiation medium [MEMα (Thermo Fisher Scientiﬁc) containing 10% FBS (Thermo Fisher Scientiﬁc), 1% penicillin/streptomycin (Thermo Fisher Scientiﬁc), 10 ng/mL bFGF (Joint Protein Central) and 5 ng/mL TGFβ (StemImmune)] for approximately 10 days to generate human myotube progenitor cells. And human myotube progenitor cells of early passages were then transduced with MyoD-ER(T) lentiviral vector and transferred to Matrigel-coated culture dishes. At 37°C, 1 μmol/L 4-OH-Tamoxifen (Sigma-Aldrich) was added into the myotube progenitor cells culture medium with 5% CO_2_ and 3%–5% O_2_ when the cells reached 70%–80% confluence. After 24 h, myotube progenitor cell culture medium was replaced with fresh hMyotube differentiation medium (high-glucose DMEM medium containing 2% horse serum) supplemented with 1 μmol/L 4-OH-Tamoxifen and then replaced with fresh hMyotube differentiation medium every 2 days. The day for the first administration of 4-OH-Tamoxifen was defined as Day 1. The mature myotubes form on Day 5 or Day 6.

### Knockdown of *FOXO3* using small interfering RNA (siRNA)

siRNA-mediated knockdown of *FOXO3* in human myotubes was performed as previously described (Wang et al., 2021a). Briefly, 25 μmol/L of negative control duplex or siRNAs against *FOXO3* was mixed with 100 μL of Opti-MEM (Gibco) and 2μL Lipofectamine™RNAiMAX Transfection Reagent (Thermo Fisher Scientific) and then added to 1-well of 12-well plates. After an 8-h incubation, the culture medium was replaced with fresh medium. The cells were collected for RT-qPCR and Western blot 72 h after transfection. *FOXO3* siRNAs were derived from a previous study and synthesized by RiboBio (China) (Wang et al., 2021a).

### SA-β-gal staining

SA-β-gal staining of hMyotube was conducted as previously described (Wang et al., 2018). Briefly, cells were fixed in 2% formaldehyde and 0.2% glutaraldehyde at RT for 5 min and stained with fresh staining solution containing X-gal at 37°C overnight after washing twice with PBS. SA-β-gal-positive cells were counted in at least three fields selected randomly by Image J software.

### TUNEL staining

TUNEL staining was performed using a TUNEL apoptosis detection Kit (Beyotime, C1088) according to the manufacturer’s instructions (Ma et al., 2022). Images were acquired using a Leica SP5 laser-scanning confocal microscope, and the percentages of positive nucleus were quantified using Image J software.

### RNA isolation and quantitative reverse transcription PCR (RT-qPCR)

Total RNA was extracted using TRIzol (Life Technologies) according to the manufacturer’s protocol. Then the GoScript™ Reverse Transcription System (Promega) was used to reverse transcribe the cDNA from 2 μg of total RNA as a template. At least three independent samples were used for RT-qPCR assay with THUNDERBIRD SYBR qPCR Mix (Toyobo) on a CFX384 Real-Time PCR system (Bio-Rad). Primer sequences used in this study are listed in Table S6.

### Western blot analysis

Western blot was performed as previously described (Wang et al., 2022). Tissues or cells were lysed with SDS lysis buffer [containing 4% SDS and 100 mmol/L Tris-HCl (pH = 6.8)] and incubated at 100°C for 10 min. To detect phosphorylated protein, RIPA lysate buffer (Beyotime, P0013B) containing a phosphatase inhibitor (Roche,4906837001) was used. A BCA Kit was used to perform protein quantification. The protein lysates were subjected to SDS-PAGE and subsequently transferred to a PVDF (polyvinylidene fluoride) membrane (Millipore). The membranes were blocked in 5% skimmed milk powder (BBI Life Sciences) in 1× TBST, incubated with the primary antibodies overnight at 4°C and with the HRP-conjugated secondary antibodies for 1 h at RT (ZSGB-BIO), followed by visualization using the ChemiDoc XRS system (Bio-Rad). The quantification was performed with Image J software. The antibodies used are listed in Table S5.

### RNA-fluorescence *in situ* hybridization (RNA-FISH)

RNA-FISH was performed as reported previously (Kishi et al., 2019). Briefly, 8-μm sections of the monkey muscle tissues embedded in the Tissue-Tek O.C.T compound were cut using a cryotome and immediately placed on the polylysine-coated coverslips. One hundred and nineteen probes used for visualization of monkey *FOXO3* pre-mRNA were designed by PaintSHOP (Hershberg et al., 2021). Briefly, sections were fixed in 4% PFA for 15 min and then treated by 0.5% Triton X-100 in 1× PBS (PBST) for 30 min. After 10 min of 10 μg/mL proteinase K (in PBST) treatment and 30 min of prehybridization at 43°C, the sections were then incubated with a hybridization mixture containing probes targeting monkey *FOXO3* pre-mRNA for at least 16 h at 43°C. The slides were washed several times and incubated for 1 h with a hybridization mixture containing Alexa488-labeled fluorescent probes at 37°C. After 10 min of Hoechst 33342 (Thermo Fisher Scientific, H3570) (in 1× PBS) incubation and several washes, the sections were mounted with VECTASHIELD Antifade Mounting Medium (Vector Laboratories, H-1000), and images were captured with a confocal laser-scanning microscope (Zeiss 900 confocal system) and analyzed by Image J software.

### Nuclei isolation and snRNA-seq on the 10× Genomics platform

Nuclei isolation was performed following a previously published protocol with some modifications (Krishnaswami et al., 2016). In brief, a piece of frozen monkey gastrocnemius muscle tissues (*n* = 16) was ground into powder separately with liquid nitrogen. This power was homogenized in 1.0 mL homogenization buffer [250 mmol/L sucrose, 25 mmol/L KCl, 5 mmol/L MgCl_2_, 10 mmol/L Tris buffer, 1 μmol/L DTT, 1× protease inhibitor, 0.4 U/μL RNaseIn, 0.2 U/μL Superasin, 0.1% Triton X-100, 1 μmol/L propidium iodide (PI), and 10 ng/mL Hoechst 33342 in Nuclease-Free water] using a freezing multisample tissue grinding system (60 Hz and 30 s per time, four times in total). Samples were filtered through a 40-micron cell strainer (BD Falcon), centrifuged at 3,000 ×*g* for 8 min at 4°C, and resuspended in PBS supplemented with 0.3% BSA, 0.4 U/μL RNaseIn and 0.2 U/μL Superasin. Hoechst 33342 and PI double-positive nuclei were sorted using FACS (BD Influx) and counted with a dual-fluorescence cell counter (Luna-FL^TM^, Logos Biosystems). Approximately 7,000 nuclei were captured for each sample with 10× Genomics Chromium Single Cell Kit (10× Genomics) version 3 following the standard protocol and then sequenced in a NovaSeq 6000 sequencing system (Illumina, 20012866).

### Bulk RNA-seq library construction and sequencing

Total RNA of monkey skeletal muscles and hMyotubes were extracted using TRIzol reagent (Thermo Fisher Scientific). RNA quality control, library construction, and high-throughput sequencing were performed for each sample as previously described (Podnar et al., 2014). Briefly, sequencing libraries were prepared using NEBNext^®^ Ultra^TM^ RNA Library Prep Kit for Illumina^®^ (NEB, USA) and individually indexed. The resultant libraries were sequenced on an Illumina paired-end sequencing platform by 150-bp read length by Novogene Bioinformatics Technology Co. Ltd.

### Processing and quality control of snRNA-seq data

Raw sequencing reads of monkey skeletal muscle were aligned to the pre-mRNA reference (Ensemble, Macaca_fascicularis_5.0) and counted using Cell Ranger (version 2.2.0) with the default parameters. The raw count matrix was filtered using CellBender (version 0.2.0) software in order to eliminate the contamination of background mRNA (Fleming et al., 2019). Seurat (version 3.2.2) object of each sample was constructed from the decontaminated matrix and nuclei with genes fewer than 200 or mitochondrial ratio more than 1% were discarded (Butler et al., 2018). Doublet removal was performed with DoubletFinder (version 2.0.3) (McGinnis et al., 2019). Afterwards, the clusters lacking specific marker genes and with relatively low gene content were also discarded.

### Integration, clustering, and identification of cell types

The following steps were processed for monkey skeletal muscle dataset as the recommended pipeline of Seurat package. First, the count matrix of each sample was normalized using the “SCTransform” function. Features and anchors for downstream integration were selected with the corresponding pipeline using the “FindIntegrationAnchors” and “IntegrateData” functions, ensuring that the calculation was based on all necessary Pearson residuals. After data integration and scaling, principal component analysis (PCA) was performed with the “RunPCA” function, and the clustering analysis was conducted with the “FindNeighbors” and “FindClusters” functions. Dimensionality was reduced with the “RunUMAP” function. Cell types were identified according to the expression levels of the classic marker genes. The marker genes of each cell type were calculated using the “FindAllMarkers” function with the cutoff of |LogFC| > 0.5 and adjusted *P* values < 0.05 using *t-*test. Marker genes for each cell type are shown in Table S2.

### Analysis of differentially expressed genes from snRNA-seq data

DEGs between different cell types of old and young groups in monkey skeletal muscle were analyzed by the function of “FindMarkers” in Seurat using Wilcoxon Rank Sum test, and were identified with the cutoff of |LogFC| > 0.25 and adjusted *P* values < 0.05 DEG lists for each cell type are shown in Table S1.

### Gene expression noise analysis

Analysis of age-relevant transcriptional noise was used to analyze the aging effects on different cell types following previous work (Angelidis et al., 2019). For each cell type, with at least 10 young and old cells, the transcriptional noise was quantified in the following manner. In brief, to account for differences in total UMI counts, all cells were down-sampled so that all cells had equal library size. To account for differences in cell-type frequency, cell numbers were down-sampled to obtain the same number of young and old cells. Next, the Euclidean distance between each cell and the corresponding cell-type mean within each age group was calculated. Transcriptional noise of each cell was then measured by this Euclidean distance. Furthermore, the Euclidean distances for each monkey and the transcriptional noise ratio between old and young groups were also calculated to indicate the change of transcriptional noise across samples during aging.

### Analysis of transcription factor regulatory network

Transcriptional regulatory network was analyzed by SCENIC (version 1.1.2.2) (Aibar et al., 2017) with default parameters. The reference of TFs was downloaded from RcisTarget (version 1.6.0) with hg19. The inference of regulatory network among the 1,824 aging-associated DEGs of monkey skeletal muscle was carried out by GENIE3 and GRNBoost in python 3.0 (Irrthum et al., 2010). Then, the enriched binding motifs and the target genes of each TF were analyzed by RcisTarget. Only target genes with high-confidence annotation were selected for the downstream analysis. The transcriptional regulatory network was visualized in Cytoscape (version 3.8.0) (Shannon et al., 2003). Core regulatory TFs of DEGs in monkey skeletal muscle during aging are shown in Table S4.

### Gene Ontology enrichment analysis

Gene Ontology (GO) enrichment analysis was performed with Metascape (Zhou et al., 2019). Representative terms were selected with the cutoff of *P* values < 0.01 and visualized with ggplot2 R package (version 3.3.2) (Wickham, 2016).

### Gene set score analysis

Public gene sets were acquired from KEGG (Kanehisa et al., 2021), MSigDB database (Liberzon et al., 2015), Aging Atlas (Guang-Hui Liu, 2021), and Regeneration Roadmap (Kang et al., 2022). FOXO3 target gene set was obtained with the SCENIC analysis described in the previous section. Gene sets were used for scoring each input cell with the Seurat function “AddModuleScore.” Differences in the scores between young and old samples were analyzed using ggpubr R package (version 0.2.4) via the Wilcoxon test. Gene sets used in this study are shown in Table S3.

### Bulk RNA-seq data processing

RNA-seq data were processed as previously described (Ma et al., 2021). In brief, to trim adapter sequence and remove low-quality reads, the raw sequencing reads were first processed with Trim Galore (version 0.6.6). Then, the cleaned reads were mapped against monkey genome reference macFas5 or human genome reference hg19 downloaded from Ensembl (Yates et al., 2020) with HISAT2 (version 2.2.1) (Kim et al., 2015), and then the mapped reads were counted with HTSeq (version 0.13.5) (Anders et al., 2015). DEG analysis was conducted with DESeq2 (version 1.28.1) in R (Love et al., 2014), and DEGs were identified as the genes with |Log_2_FC| > 0.5 and adjusted *P* values < 0.05 or *P* values < 0.05 (for monkey skeletal muscle samples). DEG lists are shown in Table S1.

### Identification of the binding motif of FOXO3 target genes

To investigate the transcriptional changes of FOXO3 target genes in skeletal muscle aging, the target genes and the binding motif sequences of FOXO3 were retrieved from the SCENIC analysis result. 

### Statistical analyses

All experimental data were statistically analyzed using PRISM software (GraphPad 8 Software). Comparisons were conducted using the two-tailed Student’s *t*-test or Wilcoxon Rank Sum test. *P* values are presented in indicated figures.

## Supplementary information

The online version contains supplementary material available at https://doi.org/10.1093/procel/pwac061.

pwac061_suppl_Supplementary_Materials

pwac061_suppl_Supplementary_Table_S1

pwac061_suppl_Supplementary_Table_S2

pwac061_suppl_Supplementary_Table_S3

pwac061_suppl_Supplementary_Table_S4

pwac061_suppl_Supplementary_Table_S5

pwac061_suppl_Supplementary_Table_S6

## Data Availability

The accession numbers for the raw snRNA-seq data and bulk RNA-seq data reported in this paper are GSA: CRA006505 and HRA002196.

## Abbreviations

snRNA-seq: single-nucleus RNA sequencing
PMF: postsynaptic muscle fiber
tSC: terminal Schwann cell
MuSC: muscle stem cell
Fib/FAP: fibroblast/fibro-adipogenic progenitor
Tendo: Tendon fibroblast
SMC: smooth muscle cell
EC: endothelial cell
Mac: macrophage
DEGs: differentially expressed genes
FOXO3: Forkhead box O3
NMJ: neuromuscular junctions
NHP: non-human primate
t-SNE: t-distributed stochastic neighbor embedding
UMAP: uniform manifold approximation and projection
CV: coefficient of variation
siRNA: small interfering RNA.

## Code availability

No specific custom code was used in this manuscript. All the codes are publicly available and the source is annotated in the text and/or in the Methods. Scripts are available upon reasonable request.
