# Supplementary material for: Single-nucleus profiling unveils a geroprotective role of the FOXO3 in primate skeletal muscle aging
Source: Protein Cell. 2022 Nov 22;14(7):499–514. doi: 10.1093/procel/pwac061 (PMC10305740; doi:10.1093/procel/pwac061)
Supplement: pwac061_suppl_Supplementary_Materials [file pwac061_suppl_supplementary_materials.pdf]

Supplemental Materials

Figure S1. Information of cynomolgus monkeys and quality control of single-nucleus RNA-seq of NHP skeletal muscle.

(A) Information of the cynomolgus monkeys used in this study.
(B) Left, PI and Hoechst double-positive nuclei were enriched by FACS. Right, representative images of nuclei after sorting. Scale bars, 50 and 10 μm (zoomed-in image).
(C) Left, bar plot showing nucleus number of each sample. Right, plot showing the Log_{10} ratio of reads per nucleus and sequencing saturation of each sample.
(D) Left, box plot showing the unique molecular identifiers (UMIs) in each nucleus. Right, box plot showing the number of genes detected in each nucleus.
(E) UMAP plot showing the distribution of clusters across all samples.
(F) Stack bar plot showing the cell proportion of each sample in 14 cell types.
(G) Bar plot showing the number of nuclei captured across cell types.

Figure S2. Single-nucleus transcriptome profiling of young and old cynomolgus monkey skeletal muscles.

(A) Box plots showing the cell proportion of each sample across different cell types. The *P* values between old and young groups were calculated using one-sided Wilcoxon Rank Sum test.
(B) Immunofluorescence staining of VWF and SMA in the skeletal muscle from young and old monkeys. Representative images are shown on the left; Scale bars, 50 and 20 μm (zoomed-in image). The numbers of VWF-positive and SMA-negative cells were quantified as fold changes in old skeletal muscles vs. in young counterparts and are presented as mean ± SEMs on the right. $n = 8$ monkeys for each group.
(C) Heatmap showing the expression profiles of genes differentially expressed in at least five cell types in the monkey skeletal muscle. Only genes with same direction of differential expression among different cell types are included.
(D) Heatmap showing the expression profiles of top 5 differentially expressed genes (DEGs) for each cell type.
(E) Heatmap showing the scaled expression patterns of hotspot genes for muscle diseases-related genes in different cell types.
(F) Network visualization of upregulated core regulatory TFs in different cell types between old and young groups. Outer nodes display different cell types and the node size positively correlates with the number of target genes differentially expressed in corresponding cell types.
(G) Representative images of negative control of *FOXO3* pre-mRNA labeling in monkey skeletal muscle by RNA-FISH. Scale bar, 20 μm.

Figure S3. Phenotypic and transcriptomic analyses of human myotubes upon silence or genetic activation of FOXO3.

(A) MyHC immunofluorescence staining showing the differentiation efficiency of $FOXO3^{+/+}$ and $FOXO3^{-/-}$ hMyotubes. Representative images are shown on the left; Scale bar, 100 μm. Data are presented as mean ± SEMs, n = 3 biological replicates for each group.

(B) Schematic showing the siRNA-mediated knockdown of *FOXO3* in WT hMyotubes.

(C) FOXO3 immunofluorescence staining in $FOXO3^{+/+}$ and $FOXO3^{2SA/2SA}$ hMyotubes. Representative images are shown on the left; Scale bar, 50 μm. The numbers of FOXO3-positive nuclei were quantified as fold changes ($FOXO3^{2SA/2SA}$ vs. $FOXO3^{+/+}$) and are presented as mean ± SEMs. n = 3 biological replicates for each group.

(D) Heatmap showing Euclidean distance of gene expression profiles between $FOXO3^{-/-}$ and $FOXO3^{+/+}$ hMyotubes.

(E) Heatmap showing the transcript levels of indicated genes in $FOXO3^{+/+}$ and $FOXO3^{-/-}$ hMyotubes by RT-qPCR. n = 3 biological replicates for each group.

Supplementary Tables

Table S1. Differentially expressed genes of bulk RNA-seq and snRNA-seq datasets.

Table S2. The marker genes of each cell type in monkey skeletal muscle.

Table S3. Gene sets used in this study.

Table S4. Core regulatory transcription factors of differentially expressed genes in monkey skeletal muscle during aging.

Table S5. List of antibodies used in this study.

Table S6. The sequences for primers used in this study.

Figure S1

A

No.	Sex	Animal Symbol	Age (years)	No.	Sex	Animal Symbol	Age (years)
1	Female	YF1	5	5	Male	YM1	5
2	Female	YF2	5	6	Male	YM2	5
3	Female	YF3	5	7	Male	YM3	6
4	Female	YF4	4	8	Male	YM4	6
9	Female	OF1	18	13	Male	OM1	18
10	Female	OF2	19	14	Male	OM2	19
11	Female	OF3	19	15	Male	OM3	20
12	Female	OF4	20	16	Male	OM4	21

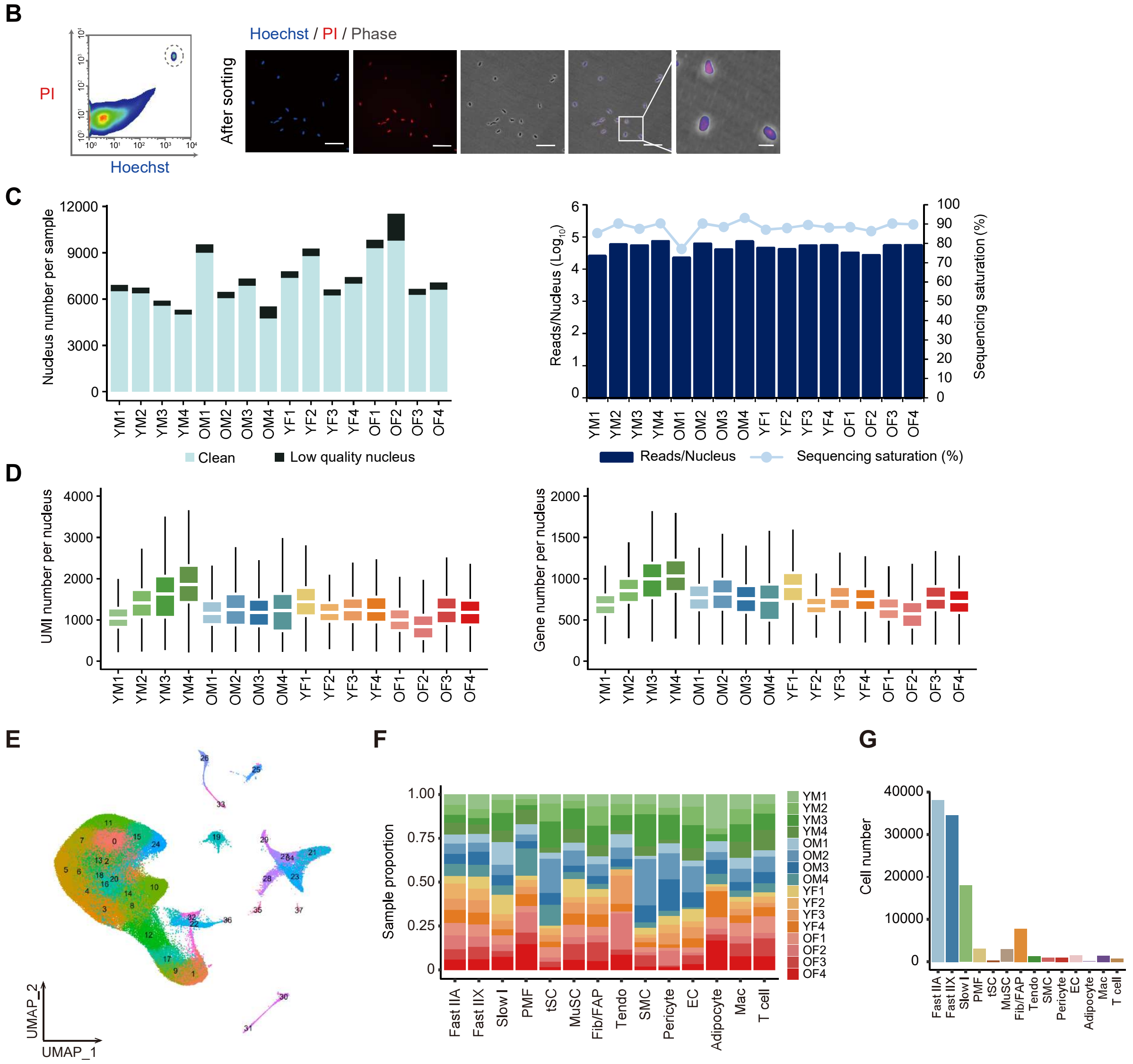

Figure S2

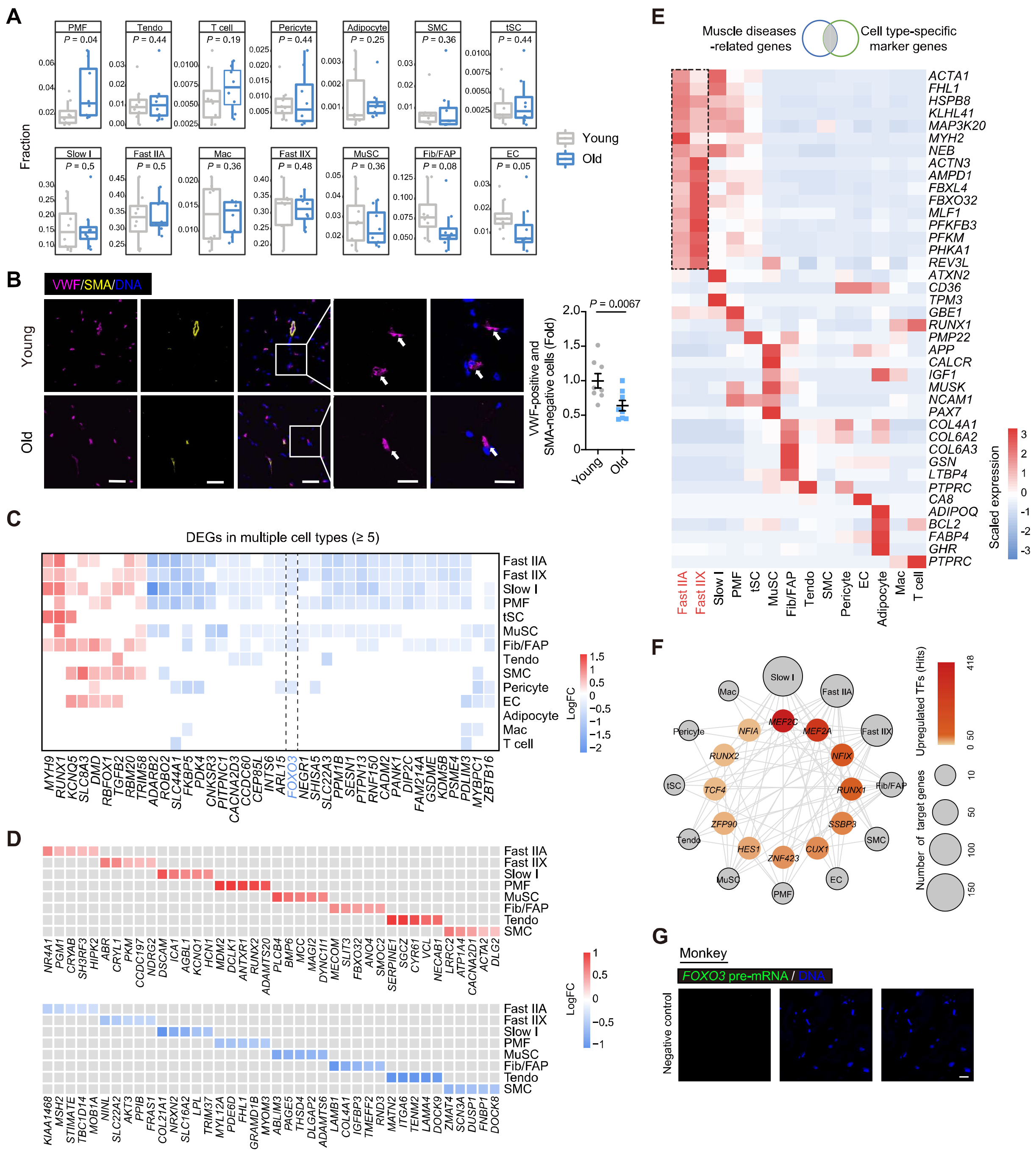
A
PMF
P = 0.04
Tendo
P = 0.44
T cell
P = 0.19
Pericyte
P = 0.44
Adipocyte
P = 0.25
SMC
P = 0.36
tSC
P = 0.44
Slow I
P = 0.5
Fast IIA
P = 0.5
Mac
P = 0.36
Fast IIX
P = 0.48
MuSC
P = 0.36
Fib/FAP
P = 0.08
EC
P = 0.05
Fraction
Young
Old
B
VWF/SMA/DNA
Young
Old
P = 0.0067
VWF-positive and SMA-negative cells (Fold)
C
DEGs in multiple cell types (≥ 5)
LogFC
D
LogFC
E
Muscle diseases -related genes
Cell type-specific marker genes
Scaled expression
F
Upregulated TFs (Hits)
Number of target genes
G
Monkey
FOXO3 pre-mRNA / DNA
Negative control

Figure S3

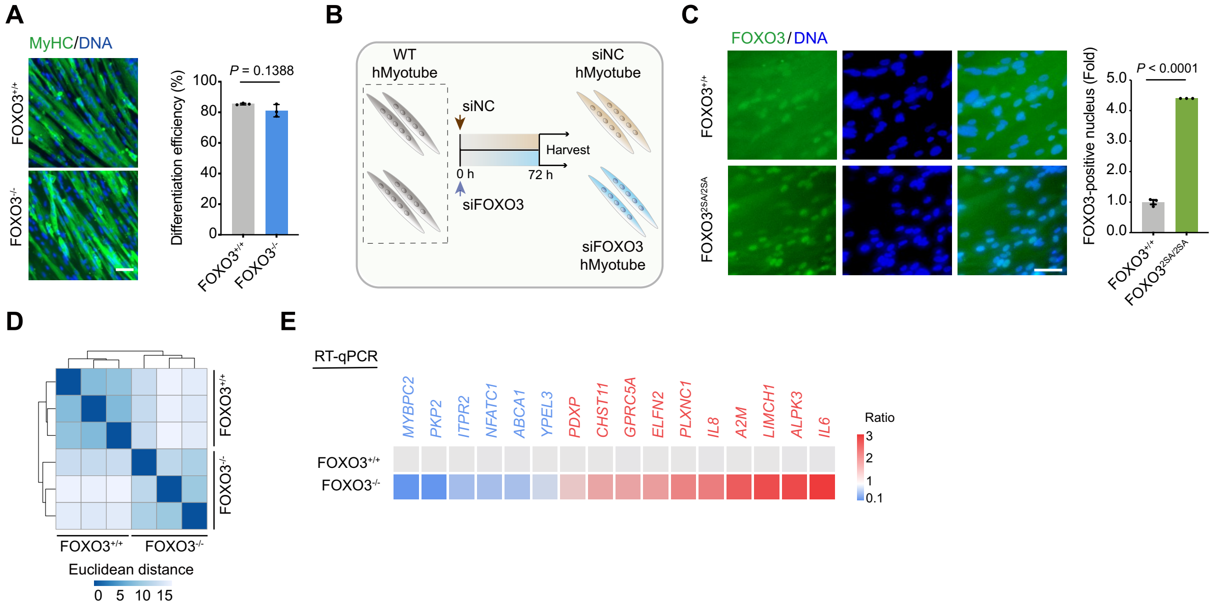
A
MyHC/DNA
FOXO3+/+
FOXO3-/-
P = 0.1388
Differentiation efficiency (%)
100
80
60
40
20
0
FOXO3+/+
FOXO3-/-
B
WT hMyotube
siNC
siFOXO3
0 h
72 h
Harvest
siNC hMyotube
siFOXO3 hMyotube
C
FOXO3/DNA
FOXO3+/+
FOXO3 2SA/2SA
P < 0.0001
FOXO3-positive nucleus (Fold)
5.0
4.0
3.0
2.0
1.0
0.0
D
FOXO3+/+
FOXO3-/-
Euclidean distance
0 5 10 15
E
RT-qPCR
MYBPC2
PKP2
ITPR2
NFATC1
ABCA1
YPEL3
PDXP
CHST11
GPRC5A
ELFN2
PLXNC1
IL8
A2M
LIMCH1
ALPK3
IL6
Ratio
3
2
1
0.1
